# Saliva as a matrix for therapeutic drug monitoring and disease biomarkers in children and adolescents

**DOI:** 10.1007/s43440-025-00732-7

**Published:** 2025-05-13

**Authors:** Matylda Resztak, Andrzej Czyrski, Joanna Sobiak

**Affiliations:** Department of Physical Pharmacy and Pharmacokinetics, Poznan University of Medical Sciences, Rokietnicka 3, Poznań, 60-806 Poland

**Keywords:** Saliva, Oral fluid, Pediatrics, Drug determination, Biomarkers

## Abstract

Saliva is a more accessible, less stressful, and less expensive biological matrix than blood, and may be applicable in therapeutic drug monitoring (TDM). Saliva concentrations reflect the pharmacologically active unbound drug. This review provides the latest information on saliva as a matrix for therapeutic drug monitoring (TDM) and biomarker determination in infants, children, and adolescents. Literature was searched up to October 2024 using the PubMed database and 64 studies were included in TDM, steroids, supplements, disease biomarkers, dentistry, genetics, and other categories. Unstimulated saliva was collected using cotton swabs or synthetic fiber rolls, as expectorated or freely flowing saliva, and stimulated by chewing on a rubber band or paraffin block. For drug determination, saliva was purified by centrifugation. Protein precipitation or extraction was rarely used. Saliva volumes for analyses were low (2.5–10 µL). Chromatographic methods and immunoassays were used for drug determination. Commercially available kits were applied for saliva hormones analysis or DNA quantification. For some antibiotics, antiepileptics, mood-stabilizers, analgesics, and immunosuppressants, saliva-plasma correlations were found. Saliva has the potential for fentanyl and prednisolone TDM in the pediatric population and for congenital adrenal hyperplasia monitoring. Salivary cortisol measurements in adolescents may play a role in sociological and psychological responses to stress, whereas in infants may reflect the depressive symptoms and higher cortisol levels of mothers. Saliva may help in diagnosing Keratoconus, pediatric-onset multiple sclerosis, sleep disorders, and quantitative behavioral difficulties. Saliva sampling depends on patient compliance. The samples may be contaminated with blood from gingival bleeding.

## Introduction

In oral physiology, saliva provides a constant rinse, is a reservoir for calcium, phosphate, and fluoride [1], and plays an important role in maintaining the integrity of dental tissues [2] and in countering pH fluctuations due to its buffering capacity [1]. Bicarbonates elevate low oral pH after meals while iron-binding protein lactoferrin or lysozyme demonstrate antibacterial capability [1]. Saliva is a major factor influencing demineralization and remineralization and, thus, the development of carious lesions [3]. Recently, enriched gums for systemic effects have been developed, which include pain killers, vitamins, alertness enhancers, or help in motion sickness removal and smoking cessation. Chewing gums increase the saliva volume and amplify the salivary buffer system, which could neutralize acidic attacks and increase dental biofilm production [1].

Saliva is mostly water, but it contains many components also found in plasma. Drugs and enzymes enter saliva through passive diffusion, reflecting the concentration in the blood. Saliva is easy to obtain, its noninvasive collection excludes skilled staff, there is less chance of adulteration, it is cheaper, and less stressful [4–6]. Only free drug enters the saliva, therefore, saliva concentrations may reflect the unbound fraction, believed to be pharmacologically active as it crosses the blood-brain barrier, for example [6, 7]. Saliva may be highly applicable in therapeutic drug monitoring (TDM), especially in infants, children, and elderly patients [4]. The Salivary Excretion Classification System divides the drugs into four classes depending on the level of intestinal permeability with protein binding [8]. Nonionizable drugs exhibit a preferable relationship between plasma and saliva concentrations [5, 9]. Saliva collection is easy and comfortable for patients, however, it might be difficult in individuals with reduced cognitive function [6]. Before introducing TDM based on saliva, plasma-saliva correlation should be defined for each drug [6].

With the advance in analytical and molecular techniques, lower drug concentrations and lower particles may be determined, e.g., in saliva. Therefore, the aim of this study is to provide the newest information on saliva as a matrix for drug analyses and biomarker determination in infants, children, and adolescents. The current study focuses on studies published mainly from 2016 to October 2024, as there is an article reviewing the studies published till 2016 [10], which focused on neonates and infants, whereas in the current study, children and adolescents were also included. Other studies on saliva as a matrix for drug determination did not include children [11–13] or were published a few years ago [14].

## Materials and methods

### Search strategy

The PubMed database was comprehensively searched in October 2024 with the combination of the terms: ‘saliva’, ‘oral fluid’, ‘pediatric’, ‘children’, ‘adolescents’, ‘infants’, ‘pharmacokinetics’, and ‘drug monitoring. ’ The search was limited to 2016–2024. The lists of studies found in the literature were searched to detect articles potentially eligible for inclusion.

### Study selection

**Fig. 1:**
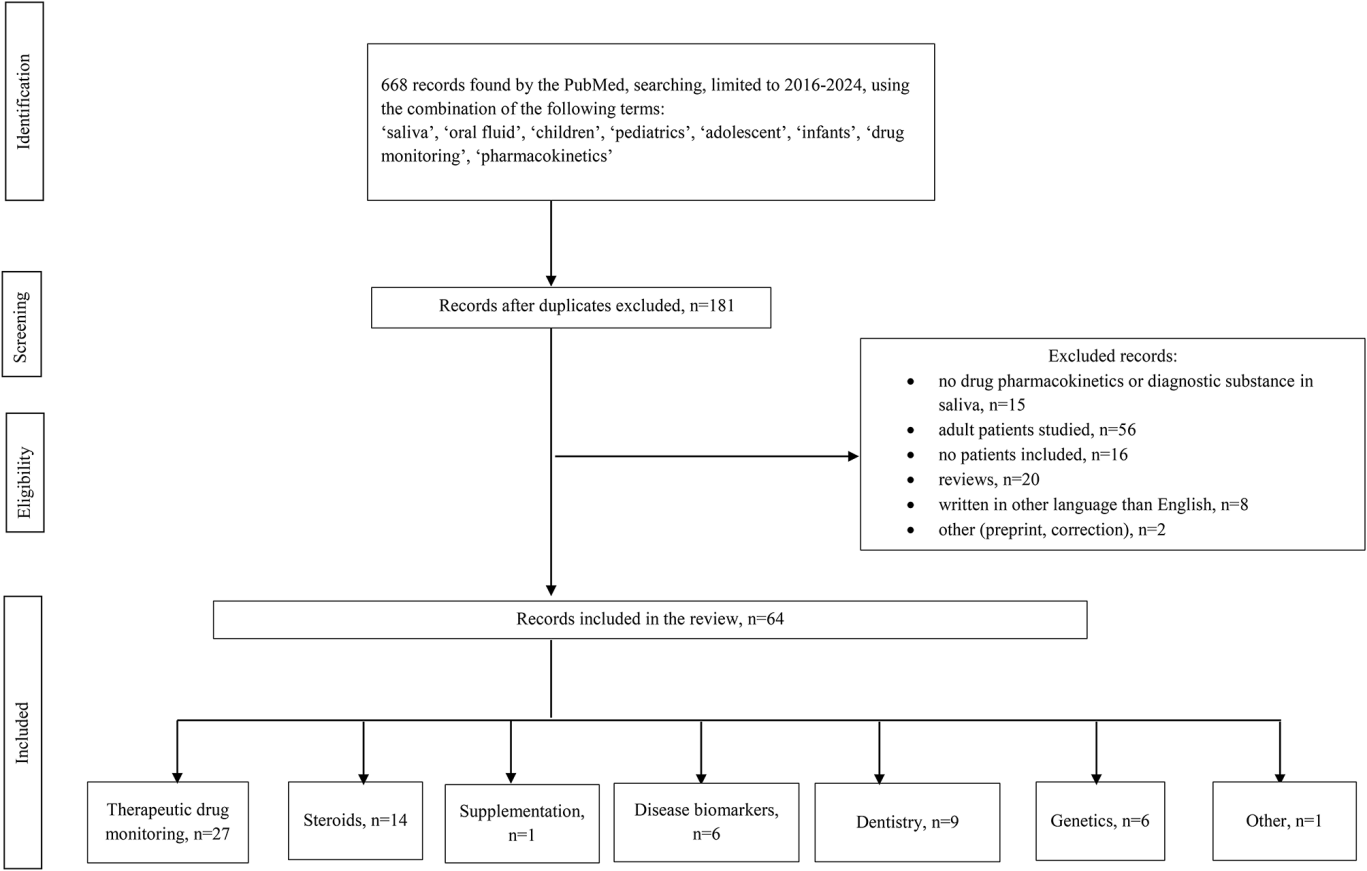
The flow diagram of article selection

The articles found were first identified, then screened, and finally checked for the eligibility. The number of 64 articles were divided into seven categories as shown on the flow diagram of article selection presented in Fig. 1.

### Inclusion criteria

Original English-written articles including infants, children, and adolescents from whom saliva samples were collected to determine drug concentrations or biomarker levels were included.

### Exclusion criteria

The articles written in languages other than English or did not contain any analytical method described or saliva measurements were excluded.

### Data analysis

Concerning the saliva application, the data were divided into the following categories: (1) TDM, (2) steroids, (3) supplements, (4) disease biomarkers, (5) dentistry, (6) genetics, and (7) others. The data were analyzed according to (1) the methods of saliva collection, (2) the analytical characteristics (samples purification and preparation, volume for analysis, analytical methods, concentration range), (3) the plasma-saliva correlations and TDM, (4) the application of saliva in disease diagnosing or as biomarker source (dentistry, genetics), and (5) other applications.

## Results

### The methods of saliva collection

Saliva may be collected using unstimulated and stimulated methods. Unstimulated methods involve collecting freely flowing saliva, such as passive drool [4, 15], into 15 mL polypropylene Falcon tubes (BD Biosciences) [16] or plastic vials (IBL-SaliCap, Germany) directly or through a straw [17, 18]. Saliva may be collected by spitting into a plastic container [19–21], directly in sterile tubes [22], or in sterile polypropylene bottles [23]. In disabled patients, the sample was taken with a plastic pipette [24]. Saliva may be collected with a mucus vacuum cleaner [25]. To maximize the simplicity of sample collection, inexpensive and readily available BD Falcon polypropylene tubes (BD Biosciences) may be applied [6]. For lactoferrin quantification, sterile Weck-Cel swabs (Beaver-Visitec International, Waltham, Massachusetts, USA), originally introduced as ophthalmic sponges, were used [26]. The Orangene^®^ DISCOVER, DNA Genotek Oragene^®^, and Oragene∙DNA saliva collection kits were applied for collecting unstimulated saliva for DNA extraction [27–29]. In a few studies, mainly in infants, saliva was expressed with a disposable syringe or drained into Salivette^®^ tubes (Sarstedt, Nümbrecht, Germany) from small sterile cotton balls or braids, which were placed in the mouth for 10–30 s or a few min to collect unstimulated saliva [30, 31]. Gesseck et al. assessed the variability of simple oral fluid collection using a sterile foam-tipped swab rinsed in phosphate-buffered saline [32]. The swab was rolled along the infant’s oral mucosal surface of the mouth, inner cheek, and tongue until saturated.

Salivette^®^ system is one of the most accessible and popular devices. It consists of a sterile swab and an exterior tube. The Salivette^®^ system was used for saliva collection in most of the studies [9, 33–49]. Other devices for saliva collection include Sorbettes^®^ (Salimetrics, State College PA) [50] or SalivaBio Infant Swab (Salimetrics, Carldbad, CA, USA). The latter consists of a durable polymer and should be placed into the cheek pouch for 90 s [51, 52] or under the child’s tongue for 1.0–1.5 min [53]. The other system for unstimulated saliva is the cellulose spear swab (EYETEC^®^, North Yorkshire, UK) [54]. Saliva may also be collected on the cellulose spear for up to 1–3 min until saturated. Longer collection was not possible in infants [54, 55].

Although some authors claim that it is the unstimulated method of saliva collection, chewing stimulates saliva flow to some extent [13, 33]. Patients are trained to chew on a swab for 2–3 min [38, 39], at least 3 min [43], or placing a synthetic fiber roll in the mouth of patients for about 5 min [49]. Other methods of saliva stimulation include chewing paraffin gum [56], a rubber band or paraffin block [2, 3, 57], or a piece of parafilm for one minute before spitting into Salivette tubes [37].

For higher volumes of saliva, stimulation with some reagents may be applied [32, 58]. Citric acid [41, 59] and sodium bicarbonate [59] are the most popular reagents. Saliva production may also be enhanced by applying citric acid cotton swabs to the buccal mucosa and subsequent collection with a Salivette pipette (Sarstedt BV, Etten-Leur, The Netherlands) [58].

In one study, patients were instructed to let saliva pool in their mouths for period five minutes while their heads were tilted forward. As they felt the need to swallow, they expectorated into a plastic re-sealable collection vial [60].

Saliva may be used directly in the diagnostics tests, such as the OraQuick^®^ test for hepatitis C virus detection [61].

The analyte adsorption on the swab may occur during sample collection. The adsorption of less than 15% is acceptable, as this is a commonly used boundary value for the precision and accuracy of quantitative analytical laboratory techniques [51]. The adsorption did not exceed 15% for gentamycin collection on SalivaBio Infant’s Swabs [51] and mycophenolic acid collection on cotton swabs in Salivette^®^ [34, 35].

### The analytical characteristics

#### Sample purification and Preparation

Since saliva is a less complex biological matrix than plasma or urine, it mostly does not need any prior sample clean-up. Centrifugation is the most commonly used technique [24, 33, 36–42, 46, 48, 50–52, 54, 55, 58, 62] for mucin removal [46] and to separate desquamated epithelial cells, food particles, and bacteria from saliva [57]. According to the analyzed literature, different centrifugation conditions were utilized for saliva sample preparation, from 2 min [58] up to 20 min [21]. The Salivette^®^ devices should be centrifuged for 2 min at 1000 g according to the manufacturer’s instruction [35], although in one study, Salivette^®^ was centrifuged for 10 min [36]. The centrifuging speed was from 2500 rpm [42] to 12 000 rpm [57] and from 1000 g [58] to 16 097.2 g [2]. Sometimes, centrifuging was at a lower temperature (4 °C [50], 7 °C [60]). After centrifugation, the collected saliva samples were generally stored at -20 °C [30, 37, 54] or at -80 °C [41, 52, 53] until analysis. Some samples were frozen in liquid nitrogen and stored in a freezer [38]. For potentiometric methods, samples were pretreated with 0.5 M NaCl as a matrix modifier [37]. One study compared two separation procedures. In the first, the saturated saliva spear was transferred to a collection tube, cut off, capped, and centrifuged, and the supernatant was frozen at -20 °C. In the second procedure, the saturated saliva swab was frozen in the tube at 20 °C and stored. The sample was centrifuged after defrosting before analysis, and the supernatant was collected. Equivalent results were obtained, however, better repeatability was observed for centrifuging right before the analysis [55].

In several studies, acetonitrile was used for protein precipitation with subsequent centrifugation [6] or evaporation to dryness [34, 35]. For protein precipitation, a mixture of 5% zinc sulfate in methanol (1:1, v/v) was also used [40, 63]. Saliva samples were also diluted with 1% aqueous solution of formic acid [55] or with nitric acid after centrifuging and protein/debris removal [64].

Some pre-analytical procedures similar to those commonly used for serum or urine were applied. Liquid extraction [45, 49, 62, 65, 66] and solid-phase extraction (SPE) [16, 32, 33, 67] were reported for salivary sample preparation. Supported liquid extraction [45] was used according to the previously published procedure [68]. On-line SPE liquid chromatography system [9, 18, 42, 45] and on-line solid-phase microextraction [54, 55] were also used.

Some specific methods of saliva preparation were also found. For example, the Bradford assay was performed for protein concentration and saliva purity assessment [4], whereas rehydration, incubation, and spinning in a filter were used for lactoferrin quantification to extract the contents of the swab [26] and lyophilization for quercetin content assessment [1]. For fluoride determination by potentiometry, saliva was buffered with total ionic strength adjustment buffer III solution [57] or modified with hexamethyldisiloxane microdiffusion method [60].

For immunoassays, no extraction was needed [25]. For DNA extraction from saliva, the samples were stored in a laboratory setting after collection until DNA extraction and gene sequencing were conducted [27]. The automated QIAamp DNA Mini Kit protocol for the QIAcube (QIAGEN, Hilden, Germany) [27] or the PrepIT-L2P kit (DNA Genotek) [28] were used.

All the above-described details of the saliva purifying and preparation procedures are presented in Table 1.

**Table 1:** The use of saliva in therapeutic drug monitoring for selected diseases in infants, children, and adolescents

Drug	Saliva collection	Saliva purifying/sample preparation	Saliva volume	Drug determination method	Concentration range or LLOQ	Studied population	TDM suitability or other application	Correlation between saliva and plasma/serum	Reference
Gentamycin	unstimulated (swab placed into the cheek pouch for 90 s)	centrifugation of swabs	a minimal sample volume of 10 µL	LC–MS/MS	LLOQ 0.056 mg/L	54 neonates	yes; a targeted peak plasma concentration was within the range of 9–11 mg/L and trough concentration < 0.8 mg/L after the third dose; TDM with four saliva samples is equal to TDM with one plasma sample	not feasible	[51]
Gentamycin	not defined	saliva mixed with 20 µL of IS and the mobile phase; centrifuged at 10°; 200 µL of upper layer transferred to the HPLC vials	200 µL	LC-MS/MS; Eclipse, C18, 100 × 4.6 mm, 3.5 μm, thermostated at 25 °C; mobile phase: 0.01% trifluoracetic acid and ACN (70:30, v/v); injection volume: 15 µL; MRM (m/z) for gentamycin: 478.3/322.1, and for solifenacin (IS): 363.0/193.0; ESI positive mode	20.0–5000.00 ng/mL, LLOQ 20 ng/mL	50 neonates (27 boys, 23 girls)	suggested level for saliva C_min_: 5 µg/mL; 5 times higher than for plasma (1 µg/mL)	the mean saliva/plasma ratio is 5–6; 5 for C_min_; 7 for C_max_	[71]
Amikacin	unstimulated (placing swabs in the cheek pouch for 90 sek)	swabs placed in collection tubes with the saturated side downwards, the unsaturated side was cut off and discarded; the tubes were refrigerated at 2–8 °C (max 12 h); for the extraction: centrifuging, freezing (-80 °C)	10 µL (minimal sample volume required)	LC–MS/MS	quantification limit: 0.010 mg/L; concentration range in the uncontaminated saliva samples: 0.044–12.1 mg/L	23 term and preterm neonates; males/females 11/12; median gestational age 28 weeks (range 25.7–29.51 weeks); median postnatal age 8.1 days (range 4.8–14.1 days)	the first report describes salivary amikacin pharmacokinetics and applicability of TDM using saliva samples in neonates with late-onset sepsis admitted to the neonatal intensive care unit; amikacin can be measured and modeled using saliva from a neonatal population; salivary concentrations of amikacin seem to reach higher concentrations in extremely premature neonates	time-dependent relationship between salivary and plasma amikacin concentrations found; TDM of amikacin using saliva samples resulted in target attainment comparable to plasma samples	[52]
Monohydroxy-carbamazepine	unsimulated (saliva was spat into a plastic container; in case of disability saliva was collected with a plastic disposable pipette)	saliva frozen after centrifugation; after thawing 200 µL mixed with IS and MeOH and centrifuged at room temperature; clear supernatant transferred to the vials	200 µL (2 mL collected from patients)	HPLC-UV; RP-ZORBAX Eclipse Plus C18 250 × 4.6 mm, 5 μm, thermostated at 40 °C; detection at λ = 254 nm; mobile phase: 45% MeOH in pure water; flow rate: 1 mL/min; IS: p-chlorobenzene acetamide; injection volume: 20 µL	2–100 mg/L	52 pediatric patients (23 males, 29 females) aged 0.67-15 years	saliva/serum ratio confirmed that saliva concentration reflects the free drug concentration in serum	high correlation between saliva and plasma; the ratio: 0.71 ± 0.17 (range 0.32–1.04; coefficient of variation 23.9%); age and sex did not have an impact on saliva/plasma ratio	[24]
Valproic acid, carbamazepine, phenytoin	stimulated (citric acid; for carbamazepine quantitation collected with sodium bicarbonate; collection without buffer for phenytoin)	no data	2 × 1 mL of saliva collected prior to the morning dose	HPLC method for valproic acid reported previously [80]	no data	30 patients	the importance of the CYP3A4*1B variant in conferring multi-drug-resistant phenotype confirmed		[59]
Rufinamide	unstimulated (saliva was expectorated into polypropylene tubes)	saliva alkalized with NH_4_OH (pH 9.25) and extracted with dichloromethane	250 µL used for the analysis [74] (2 mL of saliva collected from a patient)	HPLC-UV method developed previously [74]; Spherisorb silica column 250 × 4.6 mm, 5 μm, thermostated at 30 °C; mobile phase: methanol/dichloromethane/n-hexane 10:25:65 (v/v/v) with addition of 6 ml NH_4_OH; flow rate: 1.5 ml/min; detection at 230 nm	0.25–20.0 µg/mL, LLOQ 0.25 µg/mL [74]	26 patients (15 males, 11 females); median age: 16 years; range: 4–38 years (0–18 years: 22 patients; >18 years: 4 patients)	the method may be utilized for TDM purposes; it should not be used when suspension of the drug is used (risk of contamination by the drug residues)	saliva concentrations lower than in plasma (about 70% on average); saliva/plasma ratio 0.7 ± 0.2; the correction factor should be applied; a potential inaccuracy as the ratio ranges from 0.4 to 1.1	[66]
Levetiracetam	not defined	saliva mixed with IS prepared in 40% ACN (protein precipitation); 150 µL of the supernatant diluted with 150 µL of the mobile phase	100 µL	HPLC-UV; Luna Phenomenex C8 250 × 4.6 mm; 5 μm, thermostated at 41 °C; mobile phase: 3.5% ACN in 50 mM phosphate buffer (pH 6.0); flow rate: 1.5 ml/min; detection: λ = 205 nm (isocratic elution); IS: 5,5- diethylbarbituric acid; injection volume: 100 µL	0.48–48 µg/mL	15 children < 18 years	saliva is useful in the TDM of levetiracetam to improve the therapeutic response and patients’ compliance. The samples may be taken by parents and sent to laboratory	lower levels of levetiracetam in saliva than in plasma (average ratio: 61%). Therapeutic range: 10–50 µg/mL defined previously [82]; the corresponding saliva concentrations should be 40% (as a minimum) of this range (C_min_ 4 µg/mL)	[72]
Carbamazepine, phenytoine, phenobarbital	stimulated with citric acid; collected by commercially available saliva collecting device	saliva frozen at − 80 °C after centrifugation; after thawing saliva mixed with 200 µL of ACN comprising IS; aliquots of 10 µL used for further HPLC analysis	200 µL	HPLC-UV; Zorbax RP, C18 250 × 4.6 mm, 5 μm, thermostated at 35 °C; mobile phase: 60:20:20 (v/v/v) 50 mM potassium di-hydrogen orthophosphate buffer, ACN, and MeOH at pH 3.0; flow rate: 1.2 ml/min; detection: λ = 210 nm; IS: diazepam; injection volume: 10 µL	0.5–100 µg/mL for carbamazepine, phenytoin, and phenobarbital	116 patients (88 males, 28 females), age range 6–62 years	saliva therapeutic ranges: for carbamazepine 1.4–3.5 µg/mL, for phenytoin 1–2 µg/mL, for phenobarbital 5–15 µg/mL	mean saliva/serum ratios for carbamazepine, phenytoin and phenobarbital drug concentrations with mono- bi-, and poly-therapy were 0.37, 0.33 and 0.74; 0.48, 0.49 and 0.54; 0.41, 0.28 and 0.25, respectively; significant association with the saliva and serum levels for phenobarbital	[41]
Perampanel	unstimulated (untreated polypropylene tubes)	centrifuging after adding ACN	50 µL for total concentration; 100 µL for free (1 mL of saliva to centrifuge using ultra-30k centrifugal filter)	HPLC-MS/MS; Agela Venusil ASB C8 column 50 × 2.1 mm, 3 μm, 150 Å; thermostated at 25 °C; mobile phases: 2 mmol/L ammonium formate with 0.1% formic acid and 100% ACN with 0.1% formic acid; flow rate: 0.2 mL/min; ESI positive mode; MRM (m/z): 349.96/219.10 (perampanel) and 372.10/176.20 (trazodone; IS)	LLOQ 1.0 ng/ mL	30 adult patients, aged 16 to 60 (16 years: 1 patient, 18 years: 1 patient, 19 years: 2 patients)	proved for the first time that perampanel is measurable in saliva; potential for the clinical application of the saliva perampanel TDM	linear relationship between the total saliva perampanel concentrations and the total and free plasma perampanel concentrations	[6]
Fentanyl	Unstimulated and stimulated (a dry, soft, sterile foam-tipped swab rolled along the infant’s oral mucosal surface of the mouth, inner cheeks, and tongue until saturated)	solid-phase extraction: conditioning SPE columns with methanol and 100 mM phosphate buffer (pH 6.0); washing the applied samples with water and 100 mM acetic acid; elution with 1.0 ml 78:20:2 dichloromethane: isopropanol: ammonium hydroxide (v: v:v), evaporation with nitrogen, dissolving in 100 µl of 10 mM ammonium formate and 0.1% formic acid in water	100 µL	UPLC–MS-MS on a Xevo^®^ TQD with an ACQUITY UPLC^®^; IS: fentanyl-d5 and norfentanyl-d5	LOQ and LOD set to 10 ng/mL for fentanyl and norfentanyl	Two infants	potential to create a rapid, non-invasive method for the detection of drugs in neonates and infants using saliva; the possibility to diagnose opioid exposure and risk for neonatal abstinence syndrome prior to the onset of symptoms; the potential for the evaluation of neonate and infant pharmacokinetics, including steady-state determination, therapeutic drug monitoring, and accidental drug exposure; the measured fentanyl concentration (28 ng/mL) was within the range of a previously reported steady-state concentration	no	[32]
Tramadol	unstimulated (saliva collected in sterile polypropylene bottles)	liquid-liquid extracting with ethyl acetate, centrifuging, evaporation to dryness, dissolution of the residue in the mobile phase [75]	1 mL collected from the patient	HPLC-FLD	100–6000 ng/mL; LOQ 3 ng/mL	23 patients (16 boys, 7 girls) of 96 patients, age range: 16–18 years	correlation between plasma concentrations and main specimens (saliva and urine) in toxic dosage despite pharmacokinetic differences is relatively similar to therapeutic dosage	yes, strong correlations between plasma-saliva and plasma-urine concentrations for all studied compounds (*r* = 0.5)	[23]
Metamizole metabolites	unstimulated (oral swabs consisting of a durable polymer; the end of the collection device placed under the child’s tongue for 1.0–1.5 min)	immediately after collection, tubes centrifuged and the supernatant stored at -80 °C; 20 µL of saliva mixed with 150 µL MeOH containing 0.5% formic acid and deuterated ISs; after centrifugation, an aliquot of 2.5 µL supernatant injected into the LC-MS/MS system	20 µL (200 µL collected from the patient)	LC-MS/MS: Atlantis T3 C18 analytical column (3 mm×50 mm, 3 μm); mobile phases: water and MeOH, both supplemented with 0.1% formic acid (gradient elution); deuterated ISs (4-methylaminoantipyrine, 4-aminoantipyrine, 4-acetyl-aminoantipyrine)	LLOQ: 100 ng/mL for 4-methylaminoantipyrine and 10 ng/mL for 4-aminoantipyrine and 4-acetyl-aminoantipyrine	25 children scheduled for surgery with standard postoperative metamizole pain management, median age 46 months, range: 5–70 months; weight range: 8.7–24.8 kg	simulated sparse plasma sampling scenarios (only 1 plasma sample during the elimination phase of metamizole metabolite, approximately 4–6 h post dose) combined with rich saliva sampling (up to 6 saliva samples) resulted in pharmacokinetic parameter estimates comparable to the full plasma sampling scenario; inter- and intraindividual variability associated with saliva distribution may limit suitability of saliva as a single matrix for pharmacokinetic studies in children	saliva concentrations appeared to correlate with pharmacologically active unbound plasma concentrations; the estimated fraction distributing from plasma into saliva was very close to previously reported fraction unbound in plasma; simplified sampling scenario with up to 6 saliva samples combined with 1 plasma sample associated with similar pharmacokinetics parameter estimates as the full plasma sampling scenario	[53]
Lithium	stimulated (parafilm chewed for 1 min prior to spitting)	centrifugation; freezing (-20 °C); for potentiometric methods, samples pre-treated with NaCl 0.5 M as a matrix modifier	not defined	electrolyte analyzer based on class direct potentiometrc method and by means of atomic absorption spectrophotometer	LOD 0.03 mEq/L, LOQ 0.1 mEg/L	38 patients with bipolar disorder (adults, child and adolescents)	saliva concentrations useful for estimation of the lithium levels and checking the patients’ adherence to pharmacological treatment	higher concentrations of lithium in saliva than in serum (linear correlation, *r* = 0.767); the correlation was not age-dependent	[37]
Caffeine	unstimulated (cellulose spear swab placed in the mouth for 1–3 min until saturation)	freezing at (-20 °C); spears centrifuged before analysis; the supernatant collected	10 µL	solid phase microextraction coupled to capillary liquid chromatography with DAD detection; the method developed previously [54]	no data	47 infants (gestational age 28, range 26–30), 42.6% received intravenous caffeine and 57.4% received oral caffeine	detected salivary caffeine levels: 2.20–56.90 µg/mL (median: 16.36 µg/mL)	strong positive correlation between serum and saliva caffeine levels; saliva/serum − 0.83 (for intravenous and oral administration); for intravenous caffeine - salivary/serum − 0.727; for oral administration 0.904	[54]
Caffeine, theobromine, theophilline, paraxanthine	unstimulated (cellulose spears placed in the mouth of the patient for 1–10 min until saturation with saliva)	two situations tested with equivalent results: (1) swabs saturated with saliva spear centrifuged, the supernatant frozen (-20 °C) until analysis; (2) the saturated spear placed in the collection tube, frozen (-20 °C); centrifuged after defrosting before analysis; the supernatant collected; better repeatability with fewer impurities detected for (2); the samples acidified with 1% aqueous formic acid; diluted to 100 µL with ultra-pure water; 25 µL of diluted sample processed by solid phase microextraction	10 µL	HPLC-DAD; Zorbax SB C18-column 150 × 0.5 mm, 5 μm; detection at 275 nm; mobile phase: water and MeOH in gradient elution; flow rate: 15 µL/min	LOD 0.5 µg/mL in saliva for all analytes; method quantitation limit: 1.5 µg/mL; concentration range: 5–50 mg/L	healthy adult volunteers	caffeine saliva concentrations: 5.5–56.2 µg/mL; metabolites not found	significant correlation between serum and saliva levels: the intercept statistically equal to 0 (-2 ± 3), the slope statistically equal to 1 (0.99 ± 0.14); correlation coefficient close to 0.8	[55]
Caffeine	saliva collection system (the swab kept in the mouth for 60–90 s until saturated; the process repeated about 2 min later with the other end of the swab)	centrifuging and frozen after collection; liquid-liquid extraction with ethyl acetate (proteins precipitation); the supernatant removed and evaporated; the residue dissolved in a mobile phase	0.1 mL (the volume collected from a patient: 0.2–0.5 mL)	HPLC-DAD; Waters Symmetry C18 250 mm column; thermostated at 37 °C; detection at 280 nm; mobile phase: ACN: acetic acid: water (100:1:899); flow rate: 1.2 mL/min; the method was developed previously [76]	6.25–200 mg/L	29 infants (mean gestational age 27.9 ± 2.1 weeks)	good agreement between plasma and salivary concentrations; not significant bias; salivary samples are an appropriate alternative to blood for measuring caffeine concentrations when clinically indicated	the correlation coefficient of 0.87 between salivary and plasma concentrations; the distribution of caffeine between plasma and saliva was well described by a recirculation pharmacokinetic model	[62]
Caffeine	samples collected from the cheek internal mucus with a mucus vacuum cleaner; storage in sterile recipients	no data	0.2 mL	EMIT^®^2000 caffeine Assay	detection range: 1–30 µg/mL; therapeutic window: 8–20 µg/mL: toxic concentrations: >50 µg/mL	13 preterm infants (6 males, 7 females), mean gestational age: 32.2 ± 0.7 weeks	observed concentrations: 0.4–36.8 µg/mL	significant correlation between the salivary and serum caffeine concentrations (R^2^ = 0.76), described with an equation: Cp = 0.98xCs + 3.03 (Cs and Cp - serum and salivary concentrations); no statistical difference between the mean serum and salivary concentrations according to the administered caffeine dose	[25]
Methylphenidate and ritalinic acid (metabolite)	unstimulated (a cotton pad kept in mouth and chewed for about 2 min)	centrifugation; freezing in liquid nitrogen (-80 °C); salivary pH measurement	not defined	LC-MS/MS with appropriate kits	LOQ 1.5 ng/mL for methylphenidate; 3.5 ng/mL for ritalinic acid	36 patients with ADHD, aged 7–48 years	age-dependent correlation between methylphenidate serum and saliva concentrations; higher for children; saliva cannot replace measurements in serum; saliva may serve as an alternative matrix for TDM, especially for follow-up examinations	higher methylphenidate concentrations in saliva than in serum (saliva/serum ratio about 5) with the correlation coefficient *r* = 0.51; the correlations coefficients *r* = 0.73 and *r* = 0.63, and the saliva/serum 2.5 and 7.5 for pediatric patients and adult patients, respectively; for ritalinic acid higher concentrations in serum than in saliva (saliva/serum ratio 0.03)	[38]
Methylphenidate, dexamphetamine, atomoxetine	unstimulated (cotton wool swab placed in the mouth for at least 3 min)	centrifuging and freezing (-20 °C) within 30 min after collection; saliva vortexed with a mixture of 1-chlorobutane and ACN and centrifuged; organic layer transferred and evaporated to dryness; samples derivatized with DIB-Cl reagent	1 mL for methylphenidate and dexamphetamine, 100 mL for atomoxetine	HPLC -FLD; Phenomenex Gemini-NX C18 150 × 4.6 mm,3 μm; thermostated at 35 °C; detection at 330 nm (excitation) and 440 nm (emission); gradient elution; injection volume: 10 µL	5–160 ng/mL	16 patients: 5 females (age 10–53 years), 11 males (age 8–59 years).	saliva useful in methylphenidate and dexamphetamine determination; might be useful in testing drug abuse or patients’ compliance; tomoxetine not detected	higher saliva concentrations; highly variable saliva to serum concentration ratios for methylphenidate (4.0 ± 3.4) and dexamphetamine (2.7 ± 1.7); an inverse correlation with salivary pH (dexamphetamine more acidic than blood); no saliva-serum correlation for either drug	[39]
Methylphenidate, ritalinic acid	unstimulated	methylphenidate instable in saliva; the sample centrifuged immediately and frozen in -70 °C; protein precipitation prior to analysis	100 µL	ESI-LC–MS/MS developed previously [62]; UPLC; Synergi Polar-RP 50 × 2 mm, 2.5 μm column; data recorded by scheduled MRM, including the two most intense transitions for methylphenidate, ritalinic acid, and the ISs (methylphenidate-d9 and ritalinic acid-d10); electrospray interface in positive ion mode	1–500 ng/mL for methylphenidate; 0.25–125 ng/mL for ritalinic acid	21 children from Child and Adolescent Psychiatry, 22 patients from General Psychiatry and 16 patients from Department of Dependency	saliva methylphenidate concentrations about 4-fold higher than in blood; saliva ritalinic acid concentrations about 25-fold lower than in blood; saliva not recommended for TDM due to methylphenidate instability in saliva	significant correlations between methylphenidate and ritalinic acid concentrations in saliva and in blood for each time point for intraindividual patient comparison; high interindividual patients variability of the saliva to blood ratio for methylphenidate and ritalinic acid; methylphenidate saliva/blood ratio did not vary at the different time points; ritalinic acid saliva/blood ratio varied between the time points; no significant differences between the three patient groups for saliva/blood ratio of methylphenidate and ritalinic acid	[40]
Amphetamine	unstimulated (synthetic fiber roll, placed in the mouth for about 5 min)	liquid–liquid extraction with ethyl acetate, after evaporation of the organic solvent to dryness, the residue was derivatized with 4-(4,5- diphenyl-1 H-imidazole-2-yl)benzoyl chloride hydrochloride	1 mL [38]	HPLC-FLD	not defined	28 patient (23 boys, 5 girls), mean age, 11.3 years	amphetamine paired saliva-to-serum concentrations were highly variable and strongly affected by salivary pH, indicating that saliva is an inappropriate sampling matrix for TDM of amphetamine; Bland–Altman analysis did not support saliva as a suitable matrix for TDM	yes, strong positive linear correlation between saliva and serum concentrations (*r* = 0.628, *P* < 0.001); the saliva-to-serum concentration ratio strongly pH-dependent (*r* = 20.712, *P* < 0.001)	[49]
MMF, MPA	no data	extraction (2% HCl in water, tert-butyl methyl ether), organic layer decanted and evaporated, the residue dissolved in the mobile phase	300 µL	LC-MS/MS; Thermohypersil, BDS, C18, 50 × 4.6 mm, 3.00 μm; mobile phase: ACN and ammonium formate 10 mM + 0.05% formic acid (70%:30%); injection volume: 10 µL; run time 2 min; positive ESI mode; m/z: 434.2/114.1 for MMF; m/z: 321.2/207.1 for MPA	0.025–3.50 ng/mL for MMF; 150–20 000 ng/mL for MPA	stable kidney transplant patients, aged 4–18 years (mean 11.63 years)	saliva may be an alternative body fluid to plasma in TDM of mycophenolate mofetil and mycophenolic acid	statistically significant correlation for plasma and saliva concentrations of mycophenolate mofetil and mycophenolic acid	[65]
MPA, MPAG	unstimulated (cotton swabs)	centrifuging, freezing (-80 °C); evaporation to dryness; the residue dissolved in the mobile phase	100 µL	LC-MS/MS; Zorbax Eclipse Plus C18 (2.1 mm×100 mm, 3.5 μm thermostated at 40 °C; mobile phase: MeOH with 0.1% formic acid and water with 0.1% formic acid, in a gradient flow; flow rate: 0.5 mL/min; injection volume: 10 µL; IS: MPA-d3 in ACN for MPA; positive ESI for MPA and MPA-d3, negative ESI for MPAG; injection volume: 10 µL; m/z 321.2 > 303.05 and 321.2 > 206.9 for MPA, 495.2 > 319.25 and 495.2 > 191.05 for MPAG, and 324.1 > 306.05 and 324.1 > 209.9 for MPA-d3	2–500 ng/mL for MPA and MPAG	ten pediatric patients with nephrotic syndrome, mean age 9.1 ± 3.8 years	not studied (plasma concentrations not given)	not studied (plasma concentrations not given)	[35]
MPA	unstimulated (cotton swabs)	centrifuging, freezing (-80 °C); evaporation to dryness; residue dissolved in the mobile phase	100 µL	HLC-FLD; Zorbax Eclipse XDB C18, 150 mm×4.6 mm, 5 μm; detection at 324 nm (excitation) and 425 nm (emission); mobile phase: MeOH and 15 mM tetrabutylammonium bromide with 10 mM disodium hydrogen phosphate buffer (pH 8.5), 48:52 (v/v); IS: levofloxacin in ACN; injection volume: 20 µL	5–2000 ng/mL	two children with nephrotic syndrome, both aged 12	not studied	not studied	[34]
Aliskiren and enalapril	unstimulated	swabs kept in ice until centrifugation; freezing (-20 °C); samples purifying by solid phase extraction	no data	HPLC-MS/MS; Xselect C18 CSH column; MRM for aliskiren 552.2 m/z → 436.2 m/z, MRM for enalapril 377.3 m/z →234.2 m/z, MRM for enalaprilat 349.3 m/z → 206.1 m/z, and for benazepril (IS) 425.3 m/z → 351.2 m/z; ESI positive mode	aliskiren: 0.59–1200 ng/mL; enalapril and enalaprilate LLOQ: 0.1 ng/mL	adults (13 for aliskiren and 7 for enalapril), but the study designed with children in mind	for aliskiren, mean C_max_: 7.1 ± 8.4 ng/mL (for 0–9 h), and 7.3 ± 7.1 ng/mL (for 0–192 h); t_max_: 2.7 h (for 5 h investigation), and 6 h (for 192 h investigation period); for enalapril, C_max_: 5.5 ± 6.1 ng/mL; t_max_: 1 h	for aliskiren, saliva-to-serum ratio of k_e_: 0.84–1.31 (0 to 4.9 h of the study), altered to 0.2–4.4 h (0 to 192 h of the study); for the first 5 h after the administration the mean C_max_ in saliva about 1.4% of serum concentration, for AUC 1.5% of the value for serum; for 192 h period, AUC about 18.2% of serum; not recommended to calculate serum AUC basing on saliva AUC	[33]
Levamisole	unstimulated	collected sample stored at 4⁰C in dark for a max of 12 h prior to processing; centrifuging; freezing (-80⁰C); 100 µL of sample was mixed with 750 µI of IS; 10 min of freezing; centrifuging; supernatant injected onto the chromatographic system	100 µL	LC-MS/MS; Hypersil Gold, 50 × 2.1 mm, 1.9 μm, thermostated at 40 °C; mobile phase (gradient elution): purified water with 5% (v/v) buffer (2% formic acid/1% ammonium formate) (eluent A), ACN with 5% (v/v) buffer (2% formic acid/1% ammonium formate) (eluent B); MRM of levamisole and levamisole-D5 were 205/178 m/z and 210/183 m/z, respectively; autosampler thermostated at 10 °C; positive ionisation mode; volume of injection: 1 µL	0.1–50.0 µg/L	children	yes	median total saliva concentrations slightly higher than plasma; the saliva/plasma was acceptable (median 1.3–4.94 µg/L) except for trough concentrations where the range was vast (0.58–37.16 µg/L)	[36]
Pyrazinamide	stimulated (20 mg citric acid applied on cotton swab to the buccal mucosa; collection with a saliva pipet)	centrifuging	not defined	HPLC	concentration range: 0.585– 58.5 mg/L	*n* = 43, age 0–14 years	treatment adherence not affected significantly; the intervention-an increased caregiver reporting of medication non-adherence and caregiver reporting of difficulties they experienced with administering medication	not defined	[58]

ACN, acetonitrile; ADHD, attention-deficit/hyperactivity disorder; AUC, area under the curve; C_max_, maximum concentration; C_min_, minimal concentration; ESI, electrospray ionization mode; FLD, fluorescence detection; HPLC, high-performance liquid chromatography; IS, internal standard; LC-MS/MS, liquid chromatography-tandem mass spectrometry; LLOQ, lower limit of quantification; LOD, limit of detection; LOQ, limit of quantification; MeOH, methanol; MMF, mycophenolate mofetil; MPA, mycophenolic acid; MPA-d3, deuterated mycophenolic acid; MPAG, mycophenolic acid glucuronide; UV, ultraviolet

#### The volume for analysis

The sample volume for analysis comprised the range of 10 µL [52] to 2000 µL [4, 24, 27, 28, 59, 66], and on average was < 1 mL [9, 16, 18, 22, 25, 32, 34, 36, 41, 42, 45, 49, 51, 54, 55, 57, 62, 65, 69–72]. In one study 4.64-10.0 mL of saliva was obtained after 5 min of chewing a paraffin gum, and saliva collection was one of five tests performed [56]. The application of in-tube solid-phase microextraction allowed to the reduction of the volume from 10 µL to 2,5 µL [55].

#### The analytical methods

The most commonly used technique was high-performance liquid chromatography (HPLC) with different detectors, such as highly selective and sensitive liquid chromatography with tandem mass spectrometry (LC-MS/MS) [6, 9, 16, 18, 32, 33, 35, 36, 38, 40, 42, 45, 48, 51–53, 65, 67, 71], HPLC with ultraviolet (UV) detection [24, 41, 66, 72], HPLC with fluorescence (FLD) detection [23, 34, 39, 49] and diode array detection [54, 55, 62]. The other techniques were immunoassays [4, 17, 19, 20, 25, 26, 31, 43, 44, 46, 47, 50], and potentiometry for lithium and fluoride [2, 37, 57, 60, 69, 73]. Atomic absorption spectrophotometry was used for nickel determination [64], the colorimetric method was applied for quercetin [1], calcium and phosphate [2] determination and with Fourier transform infrared spectrometry the enrichment of deuterium in saliva samples [30] was analyzed. In several studies [9, 18, 23, 40, 42, 47, 59, 62, 66] analyses, the authors based their study on the previously published methods [55, 63, 74–80], which in the majority included plasma matrix.

The separation on the HPLC column was based on the reversed-phase mechanism. The most commonly applied analytical column was C_18_ [16, 18, 23, 24, 33–35, 39, 41, 49, 53, 55, 62, 65, 67, 71] and C_8_ column in one study [72]. The C_8_ and SB-Phenyl analytical columns were used for steroids [6, 9, 42, 45]. Long columns were used, such as 250 mm [24, 41, 62, 66, 72] as well as shorter: 30 mm [18], 50 mm [6, 16, 32, 36, 40, 53, 65], 100 mm [9, 23, 35, 42, 45, 71], and 150 mm [34, 39, 49, 55]. The grain size was 5 μm in most cases, but the smaller particles such as 1.9 μm [36, 48], 2.5 μm [40], 3 μm [6, 39, 53], and 3.5 μm [71] were also applied with the rule: the smaller grain, the shorter column. Mobile phases were mainly water/methanol or water/acetonitrile mixtures, with no more than 50% and 45% of acetonitrile and methanol, respectively. The mixtures: methanol/acetonitrile and methanol/dichloromethane/h-hexane were also used as polarity modifiers [41, 66]. The additives of the mobile phase were 0.05-2% formic acid [6, 16, 18, 32, 35, 36, 45, 53, 65], ammonium acetate [9, 16, 18, 42, 45], ammonium formate [6, 32, 36, 65], trifluoroacetic anhydride or trifluoroacetic solution (0.01%) [71], and acetic acid (0.1%) [62]. The other additives were ammonium hydroxide [66], sodium phosphate dibasic (0.01 M) [34], potassium phosphate dibasic (0.05 M) [41], tetrabutylammonium bromide [34], and orthophosphoric acid [39]. Both isocratic [23] and gradient [49] elution were used for HPLC-FLD separation. The flow ranged from 0.2 to 2 mL/min. The temperature of the column impacts the separation’s effectiveness, and in most cases, the column was heated [6, 16, 71].

Commercially available ELISA tests were used to detect saliva hormone levels [4], melatonin [19, 20], bovine lactoferrin, and human lactoferrin [26], whereas western blot was performed to investigate prolactin-induced protein expression [4] and the immunoreactive free light chains monomers and dimers [21] in saliva. Commercially available tests (“Saliva-Check Buffer GC”, “Saliva-Check Mutans GC”) were used to obtain the information on visual inspection of the hydration level, consistency of saliva, salivary pH, amount of saliva, saliva check mutants, and GC plaque indicator kit [56] as well as for assessing saliva buffer capacity [3] or testing for hepatitis C virus antibodies [61]. Proximity-enhanced extension assays with commercially available kits were applied for inflammatory biomarkers in saliva [15].

For saliva DNA quantification commercially available kits (Qubit™ dsDNA HS Assay Kit, Thermo-Fisher, Waltham, MA) for the 2.0 Qubit™ fluorometric quantification system [27] were used. A real-time polymerase chain reaction assay system was applied to test genomic saliva DNA targeting the NAT2 gene [28]. Genomic DNA was also extracted using a DNA/RNA ShieldTM collection kit (Zymo Research, Irvine, CA, USA) [81].

#### The concentration range

Only the free form of a drug, not bound to proteins, enters the saliva, therefore, the expected saliva concentrations were low [6, 7]. The lowest concentration determined in saliva was below 1 pg/mL (0.78 pg/mL) for melatonin [19, 20]. The concentrations below 1 ng/mL were determined for enalapril, enalaprilat, and levamisole (0.1 µg/L for each) [33, 36], aliskiren (0.59 ng/mL) [33], and mycophenolate mofetil (0.025 ng/mL) [65], whereas 1.0 ng/mL was set for perampanel [6]. The highest concentration (200 mg/L) in saliva was determined for caffeine [62]. The concentrations of 100 µg/mL were determined for quercetin [1], calcium [2], carbamazepine, phenytoin, phenobarbital [41] and for monohydroxycarbamazepine [24].

For steroids, the lowest analyzed concentrations were expressed in pmol/L for 17-hydroxyprogesterone (12.5 pmol/L), androstenedione (6.25 pmol/L), testosterone (5 pmol/L), 11-hydroxyandrostenedione (50 pmol/L), and 11-ketotestosterone (5 pmol/L) [45], and in pg/mL for 17-hydroxyprogesterone (11.3 pg/mL) [43]. The lowest salivary cortisol levels were equal to 0.007 µg/dL [46]. The highest concentrations were found for prednisolone (450 nmol/L) [9, 42] and 17-hydroxyprogesterone (100 nmol/L) [67].

Other units were used for lithium (mEq/L) [37], for fluoride content (ppm F [60, 69] or µg F/mL [60], mg F/mL [2], and pg F/mL [57]) as well as for water intake (mg/kg [30]).

All the above-presented details of the concentration ranges are included in Table 1.

### The plasma-saliva correlations and therapeutic drug monitoring

Plasma-saliva correlations and the potential for saliva usage in TDM in children were found for several drugs such as gentamycin [71], levamisole [36], lithium [37], monohydroxycarbamazepine [24], rufinamide [66], levetiracetam [72], carbamazepine, phenytoin, and phenobarbital [41], perampanel [6], mycophenolate mofetil and mycophenolic acid [65] and caffeine [25, 54, 55, 62], however for gentamicin opposite results were also found [51]. For amikacin, TDM using saliva samples resulted in target attainment comparable to plasma samples in neonates with late-onset sepsis [52]. In other studies, gentamycin and lithium saliva concentrations were higher than in plasma [37, 71]. For aliskiren and enalapril, the serum-saliva correlations were low and the serum/saliva ratio was variable [33]. Levamisole saliva concentrations were slightly higher due to the drug protonation, limiting its return from saliva to plasma [36]. For monohydroxycarbamazepine, rufinamide, levetiracetam, carbamazepine, phenytoin, and phenobarbital saliva concentrations were lower than in plasma which might be affected by increased salivary flow [24, 41, 66, 72]. Rufinamide saliva predose concentrations at the steady state were approximately 70% of plasma concentration as rufinamide has about 70% affinity to plasma proteins [66]. Monitoring saliva concentrations of the antiepileptic drugs provides advantages over monitoring plasma concentrations due to a significant relationship between saliva/plasma drug levels and the expected capillary/venous drug levels. The sub-therapeutic salivary concentrations were observed in most patients, whereas plasma concentrations in patients with good responses were mostly at therapeutic levels. Some polymorphisms might affect drug bioavailability and metabolism at different stages. Therefore, equal doses produced different saliva and plasma concentrations, which provide access to the central nervous system. The polymorphisms and valproic acid sub-therapeutic saliva concentrations helped to identify drug-resistant patients [59].

Saliva concentrations of metamizole metabolites appeared to correlate with unbound plasma concentrations, although inter- and intraindividual variability associated with saliva distribution may limit the suitability of saliva as a matrix for pharmacokinetic studies in children. The simulated sparse plasma sampling scenarios (1 plasma sample during the elimination phase, 4–6 h post-dose) combined with rich saliva sampling (up to 6 saliva samples) resulted in pharmacokinetics estimation comparable to the full plasma sampling scenario [53].

Significant correlations between tramadol and its metabolite concentrations in blood, saliva, and urine were found and highly potent O-desmethyl tramadol metabolite has an important role in toxicity and the likelihood of tramadol poisoning in people with extensive metabolism [23].

For amphetamine, although a strong positive linear correlation was observed in analyzed matrices in the pediatric ADHD population, the saliva/serum ratio was strongly pH dependent, which does not favor using saliva as a sampling matrix for amphetamine TDM [49].

Oral fluid has the potential in fentanyl TDM in the pediatric population although the conclusion was not based on plasma or blood concentrations [32]. Salivary concentrations may be used to assess the patients adherence to the intervention over time [58].

Salivary steroid hormones could be a noninvasive monitoring tool for disease monitoring in patients with congenital adrenal hyperplasia [43]. For androstenedione and 11-ketotestosterone strong correlations plasma-saliva correlations were found, whereas for testosterone, 17-hydroxyprogesterone, and 11-hydroxyandrostenedione the correlations were weaker [16, 45]. In children with adrenal insufficiency and congenital adrenal hyperplasia, hydrocortisone therapy was predominantly monitored by salivary profiles of 17-hydroxyprogesterone and adjusted every 3 months [43]. All the details of above-mentioned steroids determined in children’s saliva are presented in Table 2.

**Table 2:** The use of saliva in therapeutic drug monitoring of steroids in infants, children, and adolescents

Drug	Saliva collection	Saliva purifying/sample preparation	Saliva volume	Drug determination method	Concentration range or LLOQ	Studied population	TDM suitability or other application	Correlation between saliva and plasma/serum	Reference
Prednisolone	unstimulated (cotton swabs)	online solid-phase extraction liquid chromatography: conditioning extraction cartridge with ACN and water; washing the applied samples with 2% ammonium hydroxide, 2% formic acid, and 5% ACN; automatically placing the cartridge into the chromatographic system [77]	240 µL [77]	LC-MS/MS; Zorbax SB-Phenyl, 2.1 × 100 mm, 3.5 mm, thermostated at 30 °C; mobile phase: 2 mmol/L ammonium acetate:20% ACN (gradient elution); flowrate: 0.2 mL/min [77]	2–450 nmol/L (0.7–162 ng/mL)	104 children with nephrotic syndrome in remission, median age 4.6 years (range, 3.3–6.5 years)	the evidence for the possibility of prednisolone TDM trough salivary measurements; free prednisolone exposure was not associated with frequent relapses or adverse effects; population pharmacokinetic model developed for free prednisolone using salivary measurements in children with nephrotic syndrome	saliva-serum prednisolone levels relationship assumed to be the same in children as the observed in adults (not validated in children)	[9]
Prednisolone	unstimulated (synthetic swabs specifically designed to improve volume collection and increase participant compliance)	online solid-phase extraction liquid chromatography: conditioning extraction cartridge with ACN and water; washing the applied samples with 2% ammonium hydroxide, 2% formic acid, and 5% ACN; automatically placing the cartridge into the chromatographic system [8]	240 µL [8]	LC-MS/MS; Zorbax SB-Phenyl, 2.1 × 100 mm, 3.5 mm, thermostated at 30 °C; mobile phase: 2 mmol/L ammonium acetate:20% ACN (gradient elution); flowrate: 0.2 mL/min [8]	2–450 nmol/L; LLOQ 2ng/mL	77 patients; stratified by age: 6–23 months, 2–5 years, and 6–11 years; control group 6–11 years	not defined	not defined	[42]
17-hydroxyprogesterone	unstimulated (chewing a swap for at least 3 min; in children < 1 year of age, the parents/caregivers wiped the oral cavity with the swab to collect saliva)	not defined	not defined	ELISA kit for free 17-hydroxyprogesterone in saliva	11.3 pg/mL	18 patients (10 male); aged 1–68 months	owing to salivary sampling in young children blood sampling could be minimized; the collection of saliva samples can be performed at home without further stress for the children; it allows for individual titration of therapy for each patient	not defined	[43]
A4 and 17OHP	no data	solid-phase extraction, conditioning SPE columns with MeOH: isopropanol (95:5) and H_2_O), washing the applied samples with H_2_O: NH_4_OH (95:5) and MeOH: H_2_O: formic acid (20:78:2); eluting with 300 µL MeOH, evaporation with nitrogen; reconstitution in 30% MeOH	not defined	LC-MS; BEH C18 column; mobile phase: water/MeOH (gradient elution)	A4: 0.038–82.5 nmol/L; 17OHP: 0.046-100 nmol/L	39 patients (22 boys and 17 girls) median age 12 years	no clear benefit for treatment schedule (the highest dosage in the morning or evening) was established; due to the variation in individual responses, individually optimizing dose distribution and monitoring disease control at multiple time points is recommended	not defined	[67]
17OHP, A4, T, 11OHA4, 11KT	unstimulated whole saliva (passive drool) [67]	liquid and solid phase extraction: mixing and incubation of a 96-well sample plate; elution with dichloromethane, evaporation, and reconstitution; conditioning the SPE cartridge with MeOH and equilibration with water; washing the applied samples with 30% MeOH and eluting them onto the analytical column	300 µL	LC-MS/MS; C8 column; mobile phase: water/MeOH containing 0.1% formic acid and 2 mmol/L ammonium acetate (gradient elution)	17OHP: 12.5 pmol/L; A4: 6.25 pmol/L; T: 5 pmol/L; 11OHA4: 50 pmol/L: 11KT: 5 pmol/L (all LLOQ values)	78 children with congenital adrenal hyperplasia and 62 healthy controls aged 8–18 years	in children with congenital adrenal hyperplasia strong correlation between plasma and salivary concentrations of androgen hormones and correlations between plasma and salivary concentrations for 11-oxygenated-C19 androgens; the combination of salivary steroid hormones can serve as a noninvasive monitoring tool in congenital adrenal hyperplasia; it may provide a major amount of additional information and ultimately improve management and outcomes	yes, strong correlations between plasma and salivary steroid concentrations in patients with congenital adrenal hyperplasia	[45]
A4 and 17OHP	unstimulated (passive drool into polypropylene tubes)	solid-phase extraction: conditioning SPE columns with MeOH: isopropanol (95:5), washing the applied samples with H_2_O: NH_4_OH (95:5) and MeOH: H_2_O: formic acid (20:78:2); eluting with 300 µL MeOH, evaporation with nitrogen; reconstitution in 30% MeOH	500 µl	LC-MS/MS; volume of injection: 10 µL	A4: 110 − 24,700 pmol/l and 17OHP: 14–26,800 pmol/l	255 healthy pediatric and adult volunteers, aged 4–75 years	good stability of the steroids in saliva enables saliva collection by the patient at home; salivary A4 and 17-OHP display a diurnal rhythm and age‐ dependent pattern, therefore reference values for both children and adults at three time points during the day were established; the reference values support treatment monitoring of children and adults with congenital adrenal hyperplasia	not defined	[16]
A4 and 17OHP	unstimulated (passive drool)	centrifuging (desquamated epithelial cells, food particles, and bacteria separation from saliva)	not defined	commercially available ELISA kits	not defined	26 children with congenital adrenal hyperplasia; 12 children with adrenal insufficiency; 293 healthy controls	not defined	scalp hair 17-OHP strongly correlated with 17-OHP concentrations in serum (q = 0.94, *P* < 0.001) and saliva (q = 0.69, *P* = 0.009)	[44]
Cortisol	unstimulated (passive drool)	centrifuging after thawing (mucins removal)	25 µL	commercially available competitive immunoassays	range of sensitivity: 0.007–3 µg/dL	973 adolescents (mean age: 19.20 years, SD = 1.13; range: 16–23 years)	salivary cortisol one of the factors investigated on the associations between emotion socialization and psychological responses to acute psychosocial stress in late adolescence and emerging adulthood; parental emotion socialization may be an important factor influencing hypothalamic pituitary adrenocortical axis reactivity and psychological responses to stress, with important differences across gender and ethnic youth subgroups	not defined	[46]
Cortisol	unstimulated (passive drool into plastic vials, directly or through a straw)	not defined	20 uL	competitive electrochemiluminescence immunoassay ECLIA	not defined	126 primary school children and 173 adolescents from all educational streams, a 2-year interval to 221 participants, aged 9–17 years	the adolescent development of the cortisol response was related to social cognition as the transition to recursive thinking predicted an increase in the cortisol response to speech anticipation but was unrelated to the magnitude of the overall cortisol response; recursive thinking enables earlier realization of social-evaluative threat	not defined	[17]
Cortisol	unstimulated (passive drool)	not defined	300 µL [77]	ELISA method	not defined	21 children, aged 8–12 years	salivary cortisol applied to estimate physiological stress response among the elements of the symptom cluster in survivors of brain tumors; child salivary analysis revealed abnormal cortisol patterns in some participants; given the small sample size, it was difficult to draw a definitive conclusion from the study; implications for nursing: stress, sleep–wake disturbance, and fatigue symptom cluster in survivorship necessitates routine nursing assessment	not defined	[47]
Cortisol	unstimulated (Sorbettes, hydrocellulose sponge attached to a plastic shaftwhich, placed in the mother’s and infant’s mouths for 60 s)	centrifuging (at 4 °C)	not defined	enzyme immunoassay	not defined	297 infants, aged 16 and 17 months	salivary cortisol determined in mother and infant; maternal cortisol levels moderated associations between maternal depressive symptoms and infant cortisol levels; the findings in relation to environmental and biological factors may contribute to the intergenerational transmission of depressive symptoms	not defined	[50]
Cortisol	unstimulated (cotton braids, which the mother kept in the child’s mouth for 10–30 s, then drained into Salivette tubes with help of needleless syringes)	not defined	not defined	commercial chemiluminescence immunoassay technique	sensitivity of 0.16 ng/mL	404 children, aged 12 to 48 months	cortisol showed an inverted U-shaped association with four growth indexes; early life daytime cortisol levels, as a reflection of hypothalamic- pituitary-adrenal axis development, might influence growth in early infancy	not defined	[31]
Cortisol and cortisone	unstimulated (passive drool, spitting or drooling down a straw into a salicap tube)	on-line solid-phase extraction: mixing and centrifuging of a 96-deep well sample plate; clean-up and removal of saliva matrix, injection of extract onto the analytical column [79]	100 µL [79]	LC- MS/MS; positive- ionization mode	LLOQ: 0.03 µg/dL (0.8 nmol/L) for both salivary cortisol and salivary cortisone	24 healthy children	nasal administration of a newly manufactured formulation generates equivalent plasma cortisol levels to the market-available IV Synacthen formulation and provides a noninvasive test for adrenal insufficiency	not defined	[18]
Prednisolone	unstimulated (swabs)	centrifuging (10 min at 18 °C); saliva stored at -80 °C until batch analysis	10 µL	LC-MS/MS: Hypersil Gold 50 × 2.1 mm, 1.9 μm thermostated at 40 °C; injection volume: 5 µL; flow rate: 0.80 mL/min; autosampler temperature: 10 °C; IS: dexamethasone-D5; positive ionisation mode; mass transition of prednisone, prednisolone, dexamethasone and dexamethasone-D5: 359/147, 361/147, 393/373 m/z and 398/378 m/z, respectively	LLOQ for unbound prednisolone: 6.00 µg/L	45 children with steroid-sensitive nephrotic syndrome at 4 and 8 weeks of oral prednisolone treatment, median age 6 years (range 2–15 years); females/males: 15/30	saliva proved to be a reliable and patient-friendly option to determine prednisolone plasma exposure in children with steroid-sensitive nephrotic syndrome; because of the presence of interindividual variability on saliva-plasma ratio, it is recommended to collect 1 plasma sample together with 4 saliva samples to establish the individual AUC	population pharmacokinetics of unbound prednisolone in plasma and saliva in children with first onset steroid-sensitive nephrotic syndrome; unbound saliva prednisolone concentrations were correlated with unbound concentrations in plasma in children; instantaneous equilibrium between saliva and plasma with a saliva-plasma ratio of 1.30; moderate variation (41%) between subjects	[48]

17OHP, 17-hydroxyprogesterone; 11OHA4, 11-hydroxyandrostenedione; 11KT, 11-ketotestosterone; ACN, acetonitrile; A4, androstenedione; AUC, area under the curve; ELISA, enzyme-linked immunosorbent assay; IS, internal standard; LC-MS/MS, liquid chromatography-tandem mass spectrometry; LLOQ, lower limit of quantification; MeOH, methanol; T, testosterone

### Saliva in disease diagnosis or as a biomarker source

Saliva may serve as biomarker source for Keratoconus [4] and pediatric-onset multiple sclerosis [21], and may help in diagnosing sleep disorders and quantitative behavioral difficulties [19, 20], or in antibody testing for hepatitis C virus [61].

The expression of Prolactin-Induced Protein may serve as a biomarker for Keratoconus, a degenerative disease of the cornea. This glycoprotein levels are reduced in saliva, as well as in tears and plasma, and may independently or in combination with current imaging techniques, aid in screening and diagnosis of Keratoconus [4].

Saliva immunoglobulin free light chain levels are affected by the medications and may be useful for monitoring the disease activity in pediatric onset multiple sclerosis. The saliva free light chain test may discriminate between relapse and remission states also in a wider spectrum of demyelinating diseases and may help to limit frequent magnetic resonance imaging usage. The results supported the view of mucosal immunity involvement in the pathophysiology of demyelinating diseases [21].

Melatonin rhythm and its relationship with sleep and circadian parameters in drug-naive autistic children and adolescents was evaluated based on saliva sampling [19, 20]. Melatonin measurements were used to determine dim light melatonin onset, the circadian phase marker, which is defined as the time at which a salivary melatonin concentration is within 3–5 pg/mL. Melatonin was found to exhibit different patterns of secretion in autism spectrum disorder, e.g. it declines earlier with age [19]. The behavioral difficulties were closely related to sleep and circadian rhythm disturbances in autistic patients owing to melatonin determination in saliva samples [20].

Oral antibody testing may be used for rapid hepatitis C virus detection. The positive results were confirmed with RNA from venous samples. This method of testing may be found appealing but the quick result is helpful in prisons when a prisoner is being transferred or released. However, salivary testing was more expensive and less sensitive or specific than standard hepatitis C virus tests [61].

The inflammation-related biomarker patterns differed markedly between serum and saliva in children and adolescents with juvenile idiopathic arthritis. Although saliva contains many of the inflammatory molecules traditionally measured in serum and may to some extent reflect an individual’s systemic health status, saliva was not found a suitable substitute for serum when assessing systemic inflammation [15].

### Dentistry

In dentistry, saliva may be used to assess the fluoride content [2, 3, 57, 60, 69, 73], the levels of nickel, calcium, and phosphate [2, 64] and to assess the casein in sports mouthguards [56]. Moreover, quercetin may be used in chewing gums against oral *Streptococcus mutans* strains due to its antibacterial properties [1].

The study on intra-oral fluoride retention and clearance patterns from three different fluoride varnishes showed variability in fluoride intra-orally delivery, related probably to formulation differences [60]. Another study proved that applying low-fluoride formulation using the transversal technique delivered more fluoride to saliva compared to conventional toothpaste in a pea-sized amount, therefore small amount of conventional toothpaste may not be as effective as the use of a low-fluoride formulation [57]. One study compared the salivary fluoride levels following tooth brushing with amine fluoride toothpastes containing different concentrations of fluoride and evaluated the effect of rinsing with water on oral fluoride levels. Higher residual salivary fluoride concentrations with increased fluoride concentration in toothpastes and when no rinsing was performed after brushing were found, therefore, current recommendation in children with an increased caries risk should include toothpastes with > 1000 ppm of fluoride and spitting toothpaste excess with no rinsing following brushing [73]. Saliva buffer capacity and stimulated salivary flow rate were measured to monitor white spot lesions and oral health changes in response to weekly fluoride gel application after multibracket appliance treatment but no clinical effect was found [3]. The influence of children’s menu diversity on the absorption and excretion of fluoride in saliva was also determined. Based on adult volunteers, the quality of the children’s menu diversity influenced the absorption of fluoride. The results implied that the topical application of fluoride should be performed in infants fed, preferably, after the fuller diet [69].

Saliva calcium and fluoride concentrations were found to be influenced by the transient exposure to sucrose (rinse) and by biofilm accumulation in caries-free children and children with early childhood caries. For phosphate, only biofilm accumulation influenced its concentration in saliva, whereas sucrose rinse seemed to be relevant for caries-free children [2]. 

Saliva was used to monitor oral environmental changes caused by casein and sports mouthguard in vivo. Sports mouthguards have the potential to become a microbial reservoir, produce oral and systemic diseases, and cause negative changes in the oral cavities. Casein may be a preventive means in the use of sports mouthguards as its application positively influenced salivary flow, the increase of pH values, the amount of stimulated saliva as well as the buffering capacity of the athlete, improving oral health state [56].

The study on nickel release from conventional or new generations of nickel-titanium archwires showed that brackets and archwires are the source of nickel discharge from fixed orthodontic therapy. Nickel-titanium archwires might increase nickel salivary levels, whereas epoxy-coated nickel-titanium followed by copper nickel-titanium archwires might release less nickel compared to conventional nickel-titanium ones [64].

The saliva flow rate, pH value, and the density (CFU/mL) of *Streptococcus mutans* at baseline and during treatment was investigated for antibacterial properties of chewing gums containing quercetin. In young adults, quercetin was effectively released and showed an effective antimicrobial concentration in saliva, chewing gums with quercetin may help to combat caries disease [1].

### Genetics

Based on saliva samples, the variability in the treatment response of methylphenidate and isoniazid was explained as well as CYP2C19 genotype was correlated with the dose-response of lansoprazole [27, 28, 83]. Saliva was a source of DNA also for single nucleotide polymorphisms (SNPs) testing and establishing genotype-guided algorithm for acenocoumarol dosing [29].

The effect of carboxylesterase 1 (*CES1*) variants on the frequency and severity of adverse effects and dosing requirements of methylphenidate in children with ADHD were analyzed with saliva use. No associations between clinical response to methylphenidate and *CES1* genetic variation were discerned, however, several *CES1* single nucleotide variants associated with weight-based dosing and weight loss were identified. Variations in CES1 activity may impact dose requirements in children who are prescribed methylphenidate and other CES1 substrates [27].

Genomic DNA extracted from saliva was tested to describe the frequency of human SNPs in NAT2, ascribe those polymorphisms to acetylator phenotype, and correlate to serum isoniazid exposures. There were some differences in NAT2 genotype frequencies between adults and children, but they nearly disappeared if predicted acetylator phenotypes were analyzed. Saliva-based qPCR assay was not suitable to guide personalized isoniazid dosing in young children probably due to not fully matured and active NAT2 [28].

A pilot study evaluated the expression of has-miR-34a-5p and has-miR-375 in the serum and saliva of pediatric patients with migraine without aura and found that these microRNAs were expressed equally in both matrices and could be useful biomarkers of disease and drug efficacy [22].

In a clinical trial, which results have not been published yet, saliva samples will be used to check whether genotype-tailored proton pump inhibitor dosing improves asthma symptoms among children with inadequately controlled asthma and gastroesophageal reflux disease symptoms. The CYP2C19 genotype, based on saliva sampling, its influence on lansoprazole dose-response, and lansoprazole pharmacokinetic modeling are planned to be assessed. Currently, dosing of proton pump inhibitors for children is extrapolated from adult studies [83].

The genomic DNA extracted from saliva was used in a study, which hypothesized that associations between asthma outcomes and CYP3A5 polymorphisms may result from the altered metabolism of beclomethasone dipropionate. Identifying CYP3A5 variations may help individualize the dosing of the existing asthma control medications to improve asthma control [81].

For pediatric patients receiving acenocoumarol, two dosing algorithms (clinical and genetic) were developed to improve therapy results. Saliva samples were used for SNPs identification. Almost 2/3 of the variability in acenocoumarol dose requirement can be explained by clinical and genetic factors. A trend for the current guideline to overestimate the dose for patients with a homozygous variant type of genotype of VKORC1, CYP2C9*2/CYP2C9*3, and CYP2C18, and to underestimate the dose for patients with a homozygous wild-type genotype for these genes was observed. The genetic-based algorithm was the preferable one [29].

### Other applications

Saliva samples from mother and infant pairs over a 14-day period were collected to estimate the water intake cut-off value to define exclusive breastfeeding practice. In a dose-to-mother method, deuterium oxide dilution was given orally to the mother and dispersed uniformly throughout the body water and transferred to the infant through lactation. A population pharmacokinetic model of deuterium exposure in mother-infant pairs was developed and the cut-off criterion for determining exclusive breastfeeding was determined [30].

All the above-described details of using saliva in dentistry or as a disease biomarker as presented in Table 3.

**Table 3:** The use of saliva in dentistry and for selected diseases in infants, children, and adolescents

Drug	Saliva collection	Saliva purifying/sample preparation	Saliva volume	Drug determination method	Concentration range or LLOQ	Studied population	TDM suitability or other application	Correlation between saliva and plasma/serum	Reference
DENTISTRY									
Fluoride	unstimulated (wide-necked plastic flask with a capacity of 30 mL)	not defined	about 1 mL	potentiometry	0.05–0.5 ppmF	16 adult volunteers (aged 19–33 years) ingested two types of children’s meals	the quality of the children’s menu diversity influenced the absorption of fluoride; the topical application of fluoride should be performed in infants fed and following the established guidelines	the study was based on the literature data, which showed similar saliva and plasma pharmacokinetic curves of fluoride	[69]
Fluoride (retention and clearance from fluoride varnishes)	unstimulated	whole saliva (without centrifugation) and centrifuged saliva; fluoride released by acid hydrolysis and trapped in NaOH during overnight diffusion	1 mL	modified hexamethyldisiloxane microdiffusion method; fluoride combination electrode	baseline fluoride: 0.02–0.03 ppm in centrifuged saliva	18 children, aged 7–11 years	not defined; urgent need to develop efficacy guidelines for fluoride varnishes to set minimum standards and to support research on how to improve fluoride varnish formulations further	not defined	[60]
Fluoride	stimulated (chewing on a rubber band)	centrifuging to separate desquamated epithelial cells, food particles, and bacteria from the saliva	200 µL	potentiometry; fluoride ion-specific electrode coupled to an ion analyzer (total and ionic fluoride)	standard solutions for calibration: 0.125, 0.25, 0.5, 1.0, and 2.0 pg F/mL	24 volunteers, aged 8–10 years	small amount of conventional toothpaste may not be as effective as a low-fluoride formulation applied using the transversal technique	a dose-response relationship between fluoride concentration/amount of dentifrice applied and the mean AUC of salivary fluoride concentrations	[57]
Fluoride	unstimulated	not defined	not defined	fluoride ion-specific electrode	not defined	32 children grouped in: the caries-free group (mean age 72.9 months); the caries-prone group (mean age 69.6 months)	higher residual salivary fluoride concentrations with increased fluoride concentration in toothpastes and when no rinsing was performed after brushing; current recommendation for children with an increased caries risk: toothpastes with > 1000 ppm F concentration in addition to spitting excess toothpaste with no rinsing following brushing	not defined	[73]
Fluoride	stimulated (paraffin block chewed before collection)	not defined	not defined	saliva buffer capacity: commercially available test	not defined	46 multibracket appliance patients > 11 years with not less than one white spot lesion on not less than one of the four upper front teeth after debonding	no clinical effect of post-orthodontic high-dose fluoride treatment on white spot lesions and oral health changes	not defined	[3]
Calcium, phosphate, fluoride	stimulated (piece of a parafilm)	centrifuging	25 µL for calcium and phosphate, for fluoride not defined	calcium and phosphate: colorimetric method (microplate spectrophotometer), absorbance at 650 nm (calcium) and 660 nm (phosphate); fluoride: a specific electrode for fluoride ion and a potentiometer	calcium: 0–100 µg/mL; phosphate: 0–8.27 µg/mL; fluoride: 0.01–0.1 mg F/mL	28 caries-free preschoolers and 28 with early childhood caries preschoolers, aged 3–4 years at the beginning of the study	the combined effect of biofilm accumulation and sucrose rinse modified the bioavailability of calcium and fluoride in the saliva of children with early childhood caries	not defined	[2]
Nickel	unstimulated	centrifuging, protein/debris removal, dilution with nitric acid	not defined	atomic absorption spectrophotometry	accuracy limit: 0.01 µg/L	42 orthodontic patients, aged 19.79 ± 4.09 years (range: 13–27), divided into three groups of nickel-titanium, copper nickel-titanium, and epoxy nickel-titanium archwires	nickel-titanium archwires might increase nickel salivary levels, epoxy-coated nickel-titanium followed by copper nickel-titanium archwires might release less nickel compared to conventional nickel-titanium ones	not defined	[64]
Quercetin included in chewing gums	unstimulated	lyophilization, resuspension of the residues in methanol/water (80:30, v/v)	0.5 mL of methanol extracts	colorimetric method; methanol extracts reaction with aluminum chloride; absorbance at 415 nm	standard solutions: 25, 50, and 100 µg/mL	young volunteers: 18 males/18 females; mean age 16.3 years	quercetin included in experimental chewing gums could be efficiently released into the oral cavity and could promote an effective anti-caries concentration in volunteer’s saliva, without changing salivary pH values; quercetin demonstrated an effective antibacterial activity, showed a reduction of the concentration of *Streptococcus mutans* strains in saliva samples, especially after 7 days	not defined	[1]
Several salivary tests to assess oral environmental changes caused by casein and sport-mouthguard: saliva amount, pH, hydration level, consistency, saliva check mutans, GC plaque indicator kit	stimulated (paraffin gum) and unstimulated	not defined	not defined (for some tests 4.64 mL-10.0 mL)	visual inspection of saliva hydration level and consistency; pH test strip and a buffer strip; “Saliva-Check Buffer GC” and “Saliva-Check Mutans GC”; “GC plaque indicator kit”	not defined	48 active young athletes, aged 10–14 years	casein increased pH values, the amount of stimulated saliva and the buffering capacity of the athlete, improving the state of oral health in comparison to simple mouthguards; no efficacy of casein on *Streptococcus mutans* was observed; casein proved to be a preventive means in the use of sport mouthguards	not defined	[56]
Lactoferrin	unstimulated	the swabs rehydrated in sterile water, incubated at room temperature, and spun down in a cellulose acetate filter tube to extract the contents	not defined	commercial ELISA kits bovine and human lactoferrin; optical density determined at 450 nm	not defined	31 very low birth weight infants; product administration started at median 7 day after birth (range, 2–15 days) and continued for up to 30 days	levels of human lactoferrin in the saliva and stool samples, but not the plasma and urine samples, are affected by human lactoferrin intake, because the levels in saliva and stool corresponded with whether infants received expressed breast milk or donor breast milk	the supplemented bovine lactoferrin in saliva and stool declined within 24 h after administration; urine and plasma levels were constant	[26]
Prolactin-induced protein	unstimulated (passive drool)	Western blot analysis for protein concentration and purity determination saliva samples (densitometry analysis)	2 mL	commercial ELISA kits	not defined	one of the subgroups studied: 15–24 years (*n* = 8 healthy group, *n* = 19 Keratoconus group)	not defined	significant downregulation of Prolactin-Induced Protein expression in saliva of Keratoconus patients when compared to healthy controls, independent of age, sex and severity	[4]
Melatonin	unstimulated (spitting into saliva collection tubes)	not defined	not defined	salivary melatonin enzyme immunoassay kit	the first non-zero standard: 0.78 pg/mL; threshold: 4 pg/mL	autistic children and adolescents between 5 to 15 years of age (*n* = 37, mean 9.9 ± 3.02 years) and 24 children and adolescents with a normal intellectual function (*n* = 24, mean 8.42 ± 2.43 years)	different melatonin secretion pattern in children with autism spectrum disorder; a relationship of melatonin with sleep and circadian parameters	not defined	[19]
Melatonin	no data	not defined	not defined	salivary melatonin enzyme immunoassay kit	the first non-zero standard: 0.78 pg/mL; threshold: 4 pg/mL	45 autistic children between 5 and 18 years of age (mean age 10.02 years) and controls (mean age 8.83 years)	behavioral difficulties closely related to sleep and circadian rhythm disturbances in autism spectrum disorder; the advanced pattern of melatonin secretion seems to play a protective role for sleep and behavioral difficulties	not defined	[20]
Saliva immunoglobulin free light chains	unstimulated (spitting)	centrifuging	not defined	Western blot analysis; intensity (I) of the immunoreactive free light chains bands quantified by electrophoresis analysis software; the quantified intensity values in the tested samples were normalized: I_normalized_ = I_tested sample_/I_reference sample_ and defined as free light chains indices for the monomer and dimer levels (κ_M_, λ_M_, κ_D_, λ_D_); the sums of (κM + λM) and (κD + λD) were calculated and used as total free light chainf monomer and dimer indices	not defined	62 relapsing-remitting pediatric onset multiple sclerosis patients, with other demyelinating diseasesaged or non-demyelinating neurological diseases, aged 6–17 years; control group of 14 healthy children (aged 7–17 years)	In naive pediatric onset multiple sclerosis patients, the saliva levels of free light chains in relapse significantly higher than in remission; the potential of the non-invasive saliva free light chains test as a new tool for monitoring the disease activity	not defined	[21]
Hepatitis C virus	OraQuick^®^ test	not defined	not defined	OraQuick^®^ test	not defined	107 male young offenders attending a sexual health service at an English Young Offender Institutions; mean age 19.1 years (16/17 years: *n* = 18 (16.8%); 18/19 years: *n* = 39 (36.4%)	salivary rapid HCV testing acceptable among English Young Offender Inmates; not as sensitive or specific as standard HCV tests and more expensive	not defined	[61]
Inflammatory biomarkers in juvenile idiopathic arthritis	unstimulated (drooling method; unstimulated salivation rate in mL/min was calculated by dividing the collected volume of saliva by the time used (6 min))	Homogenization by pipetting before the saliva was aliquoted; frozen at the latest 30 min after sampling; stored at -80 °C until analyzed; one saliva sample was thawed at once; 50µL of each sample added to 96 well PCR plates and shipped on dry ice to the laboratory	50 µL	proximity enhanced extension assay; the commercial test included 92 high-quality assays for biomarkers related to inflammation, including groups of chemokines, chemokine receptors, cytokines, cytokine receptors, enzymes, and growth factors; data expressed as normalized protein expression (NPX) values, an arbitrary unit in a Log2 scale	average intra-assay coefficient of variability for saliva: 4%	42 participants with juvenile idiopathic arthritis (median age 13.9 years, range 11.3–15.1 years; 29 females) and 30 controls (median age 13.9 years, range 11.3–15.1 years; 22 females)	serum and salivary biomarker patterns differed markedly, suggesting that saliva may not be a suitable substitute for serum when assessing systemic inflammation in juvenile idiopathic arthritis	73 biomarkers detected in saliva; two biomarkers were unique for saliva; biomarker levels were different in serum and saliva; higher levels in serum and lower levels in saliva for most biomarkers in juvenile idiopathic arthritis versus controls and in active versus inactive disease; in saliva, several biomarkers from the chemokine family distinctly lower in the juvenile idiopathic arthritis group, and levels even lower in active disease	[15]
Methylphenidate	unstimulated (saliva collection kits)	DNA extraction from saliva; each sample was extracted following the manufacturer’s protocol	not defined	commercially available kits for DNA quantification; fluorometric quantification	not defined	99 children with attention deficit-hyperactivity disorder (mean 7.7 years; range 3–15 years)	variation in *CES1* activity may impact dose requirements in children treated with methylphenidate	not defined	[27]
NAT2 genotypes for explaining isoniazid variability	unstimulated (saliva DNA kits)	DNA extraction from saliva (modified manufacturer’s procedure): saliva samples incubation (50 °C, dry bath, 1 h); 500 µL of heated sample mixed with 20 µL PrepIT-L2P reagent and centrifuged; supernatants mixed with 100% ethanol before pelleting DNA by further centrifugation; DNA pellets washed with 70% ethanol; dried; resuspended with 50 µL of TE solution (10 mM Tris, 1 mM EDTA, pH 8)	not defined	qRT-PCR	not defined	50 children < 15 years of age starting treatment for drug-susceptible tuberculosis (median 2.3 years, range 0.3–14.8 years)	saliva based qPCR assay was not fieldable to guide personalized isoniazid dosing in young children because of not full NAT2 maturation and activity	for C_max_ and AUC_0 − 12_ no statistically significant relation either differences across individual loci or between predicted acetylator phenotype	[28]
CYP2C19 genotyping for lansoprazole dosing	not defined	not defined	not defined	not defined	not defined	children aged 6–17 years old with clinician-diagnosed asthma and mild co-morbid gastroesophageal reflux disease symptoms	designed clinical trial; the results not published yet	pharmacokinetic modeling of the oral lansoprazole-designed	[83]
Hsa-miR-34a-5p and hsa-miR-375 expression	saliva collected in sterile tubes	extraction of total RNA with commercial kit: samples lysis with 5 volumes Lysis Reagent (5 min, room temperature), chloroform (200 µL) added; the mixture vortexed, kept at room temperature for 5 min, centrifuged (15 min, 4 °C); the upper (aqueous) phase collected; 1.5 volumes of 100% ethanol added; the solution passed through spin column in sequential 700 µL aliquots; total RNA binded to the membrane; phenol and other contaminants washed away; RNA washed once with 80% ethanol, dried for 2 min by centrifugation, dissolved in 15 µL of RNase-free water	200 µL (2 mL from patients)	termed looped primer RT-PCR for micro RNA quantification, following manufacturer’s protocol: 10 ng of total RNA subjected to reverse transcription polymerase chain reaction (for miRNA targets and endogenous control; thermocycling conditions: 30 min at 16°C, followed by 30 min at 42°C, 5 min at 85°C and 5 min at 4°C); qRT-PCR performed using commercial kit according to the manufacturer’s protocol; miRNA quantitative PCR results normalized to U6 as control levels’; the reactions performed in triplicate for each patient; incubated in optical 96-well reaction plates; thermocycling conditions: 95 °C for 10 min, and 40 cycles of 15 s at 95 °C, followed by 1 min at 60 °C; the average values of the cycle threshold (Ct) of the reactions in triplicate determined; the comparative Ct method adopted, U6 used as endogenous control; the relative expression of miRNAs tested normalized by the average values of CtU6 and the Ct target miR–CtU6; the difference plotted as 1/∆ct × 10 directly	not defined	24 patients with migraine included (12 females, 12 males), aged 4–17 years with mean 11 ± 3.467 years	hsa-miR-34a-5p and hsa-miR-375 could be considered a biomarker of disease and of drug efficacy in young subjects with migraine without aura; saliva could be used to monitor these biomarkers	saliva and serum frozen fractions obtained from all enrolled subjects with migraine (treated and untreated) constitutively expressed both hsa-miR-34a-5p and hsa-miR-375, without difference respect to age or gender. comparable levels of hsa-miRs tested in blood and saliva; saliva mirrors the serum expression profile and can be used by itself, instead of blood; the hsa-miR-34a-5p and hsa-miR-375 expression in saliva of migraine subjects enrolled in untreated subjects respect to both biological fluid, without difference respect to age or gender; a significant decrease of about 50% for hsa-miR-34a-5p in both serum and saliva of treated patients compared to untreated patients, without difference respect to age or gender; in both serum and saliva, a significant decrease (about 50%) of hsa-miR-375 levels in treated patients compared to untreated ones, without difference with respect to age or gender	[22]
Single-nucleotide polymorphisms of CYP3A5	unstimulated (saliva or buccal swabs-younger children with a sterile DNA/RNA collection kit pre-filled with a DNA-stabilizing agent)	genomic DNA isolated using commercial kits	2 mL	CYP3A5 allele status determined from 10 ng of genomic DNA using the single-nucleotide polymorphism genotyping enzyme and probes for rs776746, CYP3A5*1D/rs15524, *5/rs55965422, *6/rs10264272, *7/rs41303343, *8/rs55817950, *9/rs28383479, K208K/rs10264272, CYP3A4*22 (rs35599367) allele status was a C_59013445_10	not defined	166 children with persistent asthma, median age 8.35 years (range 2–18 years)	detection of CYP3A5*3/3, CYPA35*1/*3, and CYP3A5*1/*1 could impact inhaled steroid treatment strategies for asthma in the future	CYP3A5*3/*3 genotype associated with improved asthma outcomes; the effect was not specific to the prescribed inhaled corticosteroid, but appeared to be a general effect of CYP3A5 (and 3A4) deficiency, potentially involving the maintenance of a more “normal” cortisol level post-inhaled corticosteroid use, as opposed to a direct effect on inhaled corticosteroid pharmacokinetics	[81]
Acenocoumarol	unstimulated (saliva DNA kits)	no data	no data	SNPs genotyped: VKORC1 rs9934438, CYP2C9 rs1799853 and rs1057910, CYP4F2 rs2108622, CYP3A4 rs35599367 and rs2740574, and CYP2C18 rs1998591	no data	80 patients	polymorphisms in flanking VKORC1, CYP2C9 and CYP2C18 all increase the variability. It was possible to explain ca. 62% variability, which may improve the therapy		[29]
Water intake cut-off value	unstimulated (small sterile cotton balls placed in the mouths of the mothers and infants for a few min; saliva expressed from the cotton ball with a disposable syringe)	centrifuging	not defined	Fourier transform infrared spectrometry	limit of quantitation: 20 mg/kg; limit of detection: 6 mg/kg	121 healthy infants, aged 2.5–5.5 months exclusively breastfeeded	the cut-off value of the posterior distribution of non-milk water intake: 86.6 g/day	not defined	[30]

AUC, area under the curve; C_max_, maximum concentration; ELISA, enzyme-linked immunosorbent assay; LLOQ, lower limit of quantification; qPCR, quantitative real-time polymerase chain reaction; qRT-PCR, quantitative real-time polymerase chain reaction; SNPs, single nucleotide polymorphisms

## Conclusions

TDM in children based on saliva sampling has been proven possible for several drugs. For some antibiotics, antiepileptics, mood-stabilizers, analgesics, and immunosuppressants the correlations between saliva and plasma or serum were found. For fentanyl and prednisolone, oral fluid has the potential to be used in TDM in the pediatric population and salivary steroid hormones could be a noninvasive monitoring tool for disease monitoring in patients with congenital adrenal hyperplasia. Salivary cortisol measurements were found to play a role in sociological and psychological responses to stress in adolescents, whereas in infants salivary cortisol reflected the depressive symptoms and higher cortisol levels of mothers. On the other hand, TDM based on saliva measurements is not possible for gentamycin. For some drugs, the correlations of drug concentrations in serum and saliva were low and the serum/saliva ratio was variable (aliskiren and enalaprile), or no clear correlation was found (methylphenidate and dexamphetamine).

Saliva may also provide some factors, which are biomarkers and may play a role in disease diagnosis for such disorders as Keratoconus, pediatric onset multiple sclerosis, sleep disorders and quantitative behavioral difficulties, or in antibody testing for hepatitis C virus.

There are numerous advantages to saliva sampling in children as it might be convenient, especially for patients with needle fear [37] or may be applied to newborns without causing stress [31]. Children and their parents might also be instructed to collect saliva at home [48] what eliminates further stress for the children, minimizes blood sampling and allows for individual titration of therapy for each patient [43]. If sampling is less stressful, the steroid levels are less likely to be affected by specimen sampling. No hospitalization is required for androgens measurements in saliva, and with the use of an ambulatory blood pressure monitor what minimizes participants’ burden [67]. Based on the saliva analyses, some recommendations were given, e.g. for autistic patients, for whom sleep and circadian rhythm disturbances should be assessed daily and included in behavioral and pharmacological management strategies in autistic children and adolescents. These assessments include melatonin measurements performed in saliva [20]. Salivary glucocorticoid measurements were used in new non-invasive tests for adrenal function testing development, which may be conducted globally in community and outpatient settings, with potential cost savings, and reduced healthcare burden to patients [18]. For amikacin, the developed pop-PK model, based on saliva concentrations in a neonatal population, could be used to simulate and predict salivary amikacin distributions within the population. It allows for the assessment of dose adequacy or the evaluation and optimization of multiple TDM sampling strategies [52].

Saliva has a potential to be used in toxicology screening e.g. in the case of fentanyl and diagnosing opioid exposure and risk for neonatal abstinence syndrome prior to the onset of symptoms [32] as well as in the case of tramadol, for which the correlation between plasma and saliva concentrations in toxic dosage was found [23]. For amphetamine, saliva was not found to be appropriate matrix for TDM, however, it may be useful for conforming and excluding the presence of amphetamine [49].

Saliva sampling mainly depends on patient compliance [44]. Some difficulties may occur in obtaining the appropriate sample quantity if the collection was by spitting into the tubes a few times a day [20, 40]. The contamination of saliva samples with blood from gingival bleeding [40] is another problem. The compliance with treatment is difficult to control when hospitalization is not required [67]. Moreover, more research is required to define the target values for saliva drug concentrations or biomarker levels to benefit fully from saliva.

The study has some limitations. Firstly, we searched only the Pubmed database. Secondly, we included only English written studies.

## Data Availability

No datasets were generated or analysed during the current study.

## Abbreviations

17OHP: 17-hydroxyprogesterone
11OHA4: 11-hydroxyandrostenedione
11KT: 11-ketotestosterone
CAN: Acetonitrile
ADHD: Attention-deficit/hyperactivity disorder
A4: Androstenedione
AUC: Area under the curve
C_max_: Maximum concentration
C_min_: Minimal concentration
ELISA: Enzyme-linked immunosorbent assay
ESI: Electrospray ionization mode
FLD: Fluorescence
HPLC: High-performance liquid chromatography
IS: Internal standard
LC-MS/MS: Liquid chromatography with tandem mass spectrometry
LLOQ: Lower limit of quantification
LOD: Limit of detection
LOQ: Limit of quantification
MeOH: Methanol
MMF: Mycophenolate mofetil
MPA: Mycophenolic acid
MPA-d3: Deuterated mycophenolic acid
MPAG: Mycophenolic acid glucuronide
qPCR: Quantitative real-time polymerase chain reaction
qRT-PCR: Quantitative real-time polymerase chain reaction
SNPs: Single nucleotide polymorphisms
T: Testosterone
UV: Ultraviolet
TDM: Therapeutic Drug Monitoring
